# Indobufen-based dual antiplatelet therapy in patients undergoing PCI: a systematic review and meta-analysis

**DOI:** 10.3389/fphar.2026.1821883

**Published:** 2026-07-24

**Authors:** Yiheng Liu, Bin Liu, Weiran Dai

**Affiliations:** 1 Department of Cardiology, The Second Affiliated Hospital of Chongqing Medical University, Chongqing, China; 2 Department of Cardiology, West China Hospital, Sichuan University, Chengdu, China

**Keywords:** aspirin, cardiovascular outcomes, dual antiplatelet therapy, indobufen, major adverse cardiac events, meta-analysis

## Abstract

**Background:**

Conventional dual antiplatelet therapy (DAPT) post percutaneous coronary intervention (PCI) comprises aspirin and a P2Y_12_ inhibitor. However, aspirin increases gastrointestinal and bleeding risks. Indobufen, a reversible COX-1 inhibitor, may offer a safer alternative.

**Objectives:**

To evaluate whether replacing aspirin with indobufen in DAPT provides comparable efficacy and improved safety in patients undergoing PCI.

**Methods:**

Six databases were systematically searched until 24 February 2026. The primary outcomes were major adverse cardiovascular events (MACE) for efficacy and bleeding events for safety. Subgroup analyses, particularly for acute coronary syndrome (ACS) patients, were conducted.

**Results:**

Twelve studies involving 7,928 patients were included. Indobufen demonstrated comparable efficacy to aspirin in preventing MACE (OR = 0.89, 95% CI 0.73–1.10, *P* = 0.284). Crucially, indobufen significantly reduced bleeding events compared to aspirin (OR = 0.51, 95% CI 0.35–0.74, *P* < 0.001). Subgroup analyses across ACS status and specific P2Y_12_ inhibitors (clopidogrel or ticagrelor) consistently supported these findings.

**Conclusion:**

For patients undergoing PCI, an indobufen-based DAPT regimen appears to reduce bleeding risk while offering comparable MACE prevention relative to conventional aspirin-based DAPT. However, these findings should be interpreted cautiously due to existing limitations.

## 1 Introduction

Coronary heart disease (CHD) - encompassing both acute coronary syndrome (ACS) and chronic coronary syndrome (CCS) - remains a major and growing global health burden (Tsao et al., 2023). Many patients with CHD require antiplatelet therapy for secondary prevention, and dual antiplatelet therapy (DAPT) is routinely prescribed after percutaneous coronary intervention (PCI) to reduce thrombotic events (Byrne et al., 2023). However, intensified antiplatelet therapy may increase bleeding risk, thereby limiting treatment tolerability and adherence. Aspirin, an antiplatelet agent that inhibits the thromboxane A2 (TXA2) pathway, is a cornerstone of antiplatelet therapy and has been shown to reduce thrombotic events (Baigent et al., 2009). Nevertheless, the clinical use of aspirin is often limited by gastrointestinal adverse reactions, including gastric ulcers and erosions, which may result from its inhibition of prostaglandin synthesis—a key protective mediator in the gastric mucos (Floyd and Ferro, 2014). Additionally, a subset of patients exhibits hypersensitivity to aspirin, manifesting as bronchospasm, urticaria, angioedema, or even anaphylaxis, ultimately leading to poor medication adherence (Galli et al., 2024). These concerns may lead to treatment interruption which may increase ischemic risk, particularly in patients undergoing PCI for ACS or CCS who are treated with DAPT.

While P2Y_12_ inhibitor monotherapy has emerged as a major strategy to mitigate bleeding risks, optimal ischemic protection post-PCI often still requires dual-pathway inhibition (Mehran et al., 2019). Therefore, substituting aspirin with a safer alternative antiplatelet agent or a low-dose oral anticoagulant may provide a vital therapeutic strategy. Indobufen, an oral antiplatelet drug, can inhibit platelet activation, adhesion, and aggregation, and its antiplatelet effect is achieved by reversible inhibition of cyclooxygenase-1 (COX-1) while minimally affecting prostaglandin synthesis. Indobufen inhibits thromboxane production and cyclooxygenase-dependent platelet aggregation with minimal affecting prostaglandin production (Wiseman et al., 1992). In contrast to the irreversible effect of aspirin, which lasts for about 7 days, the antiplatelet effect of indobufen is transient, and platelet function returns to normal within 24 h after discontinuation (Wiseman et al., 1992). Previous studies have compared the incidence of cardiovascular and cerebrovascular events and drug-related adverse reactions over a defined follow-up period between patients receiving indobufen monotherapy and those receiving aspirin monotherapy. Most studies suggest that indobufen may reduce aspirin-associated hypersensitivity and gastrointestinal symptoms and provide comparable antiplatelet efficacy (Cavalcante et al., 2025). However, these findings from monotherapy cannot be directly extrapolated to DAPT, where concomitant P2Y_12_ inhibition may alter both thrombotic protection and bleeding risk.

Nevertheless, it remains unclear whether indobufen can replace aspirin when combined with a P2Y_12_ receptor antagonist in DAPT. To date, only a few studies have compared the efficacy of indobufen versus aspirin in patients undergoing PCI. The OPTION trial is the largest multicenter randomized controlled trial (RCT) conducted to date that compares indobufen versus aspirin-based DAPT in patients undergoing PCI (Wu et al., 2023). Enrolling a population with troponin-negative CHD, the study demonstrated that indobufen offers a favorable efficacy and safety profile. However, the OPTION trial’s eligibility criteria explicitly excluded patients with biomarker-positive ACS and those with known aspirin intolerance. Consequently, the efficacy and safety of an indobufen-based DAPT regimen in patients with ACS and/or aspirin intolerance remain uncertain. Recently, a real-world retrospective study in patients undergoing PCI suggested that, compared with aspirin-based DAPT, an indobufen-based DAPT regimen was associated with a comparable risk of adverse cardiovascular events but a significantly lower risk of bleeding (Dai C. et al., 2024). Nevertheless, it was an observational study and enrolled only a limited number of ACS patients. However, high-quality evidence supporting indobufen as a substitute for aspirin in DAPT, particularly in ACS, remains limited.

To address this evidence gap, we conducted a meta-analysis to evaluate the efficacy and safety of indobufen compared with aspirin-based DAPT in patients with CHD undergoing PCI. We additionally performed analyses in patients with ACS to inform clinical decision-making in this population.

## 2 Materials and methods

### 2.1 Literature search strategy

This systematic review and meta-analysis was conducted and reported in accordance with the Preferred Reporting Items for Systematic Reviews and Meta-Analyses 2020 statement (Page et al., 2021). Ethical approval and patient consent were not required for this study, as it is a systematic review and meta-analysis of previously published literature. A comprehensive literature search was conducted in PubMed, Web of Science, EMBASE, the Cochrane Library, China National Knowledge Infrastructure (CNKI), and the Wanfang Database from their inception until 24 February 2026. The search was performed by two independent investigators (YL and BL) and was restricted to publications in English and Chinese. The search strategy combined controlled vocabulary terms and free-text keywords related to indobufen, coronary heart disease, myocardial infarction, acute coronary syndrome, and percutaneous coronary intervention. The following search terms were used (“indobufen” OR “Ibustrin”) AND (“percutaneous coronary intervention” OR “PCI” OR “coronary heart disease” OR “CHD” OR “myocardial infarction” OR “MI” OR “acute myocardial infarction” OR “AMI” OR “acute coronary syndrome” OR “ACS”). The search strategy was adapted for each database as appropriate. The detailed search strategy is provided in Supplementary Table S1. The reference lists of all retrieved articles were manually screened to identify any additional potentially eligible studies. In cases where multiple publications pertained to the same study, the most recent version was selected for inclusion and analysis.

### 2.2 Literature selection

Following the initial search, articles that fulfilled the inclusion criteria were selected through a comprehensive full-text review and were included for subsequent data analysis. The inclusion criteria are as follows: 1) RCTs or retrospective studies; 2) Patients diagnosed with CHD (including stable angina, unstable angina, or myocardial infarction) who underwent PCI; 3) The experimental group received DAPT consisting of indobufen plus a P2Y_12_ receptor antagonist following PCI, while the control group received standard DAPT with aspirin and a P2Y_12_ receptor antagonist; 4) Studies reported at least one primary or secondary outcome. Studies were excluded according to the following criteria: 1) Conference abstracts for which full-text articles were unavailable; 2) Studies with incomplete data or without access to the original datasets; 3) Studies did not involve patients with CHD; 4) Studies did not report relevant outcome events.

### 2.3 Data extraction and quality assessment

Two reviewers (YL and BL) independently screened titles/abstracts and full texts, extracted data, and assessed methodological quality in duplicate. Disagreements were resolved by consensus or, when necessary, by consultation with a third reviewer (WD).

The data extracted were as follows: author, type of participant, study time, gender, age, intervening measures (drug name, initial time, dosage, frequency, and follow-up time), primary outcomes (incidence of MACE, bleeding events and gastrointestinal symptom during follow-up), and secondary outcomes (incidence of myocardial reinfarction, repeated revascularization, cardiovascular death and stroke during follow-up). Of the multiple publications identified for a single study, only the most recent one was included for further analysis. When necessary, the corresponding authors were contacted to obtain additional or clarifying information. For the randomized controlled trials (RCTs), the risk of bias was evaluated using the Cochrane Risk of Bias 2 (RoB 2) tool, and each domain was categorized as ‘low risk,’ ‘high risk,’ or ‘some concerns.’ For the retrospective observational studies, the methodological quality was assessed using the Newcastle-Ottawa Scale (NOS), which evaluates study quality based on selection, comparability, and outcome. Studies with an NOS score of ≥7 stars were considered as high quality (Sterne et al., 2019; Stang, 2010). Overall, the included studies demonstrated satisfactory quality, with all observational studies rated as high quality and the RCTs exhibiting a low to moderate risk of bias (Supplementary Table S2 AND Supplementary Figure S1). To enhance the transparency and reproducibility of our findings, the specific definitions of MACE, bleeding, and gastrointestinal symptoms for each included study are summarized in Supplementary Table S3.

### 2.4 Protocol and registration

The review protocol was registered in PROSPERO, the International Prospective Register of Systematic Reviews, under the registration number CRD420261409768.

### 2.5 Statistical analysis

All statistical analyses were conducted using Stata/SE version 15.1 (Stata Corp., College Station, Texas). Pooled effect estimates are reported as crude odds ratios (ORs) with their corresponding 95% confidence intervals (CIs). Heterogeneity across studies was evaluated with the *I* ^*2*^ statistic, with values of 0%–25%, 25%–50%, 50%–75%, and 75%–100% indicating negligible, low, moderate, and significant heterogeneity, respectively. A fixed-effects model was applied when heterogeneity was not significant (*I* ^*2*^ < 50% and *P* > 0.10). Otherwise, a random-effects model was employed, and potential sources of heterogeneity were further explored. Publication bias was assessed using funnel plots and Egger’s test, with *P* > 0.05 indicating no significant bias. To explore potential sources of heterogeneity within the CHD population, subgroup analyses were performed based on the disease population (ACS vs. overall CHD), study design (RCT vs. non-RCT), follow up time and type of concomitant P2Y_12_ inhibitor. We set the *P* value <0.05 as the statistically significant level.

## 3 Results

### 3.1 Literature search results

**FIGURE 1:**
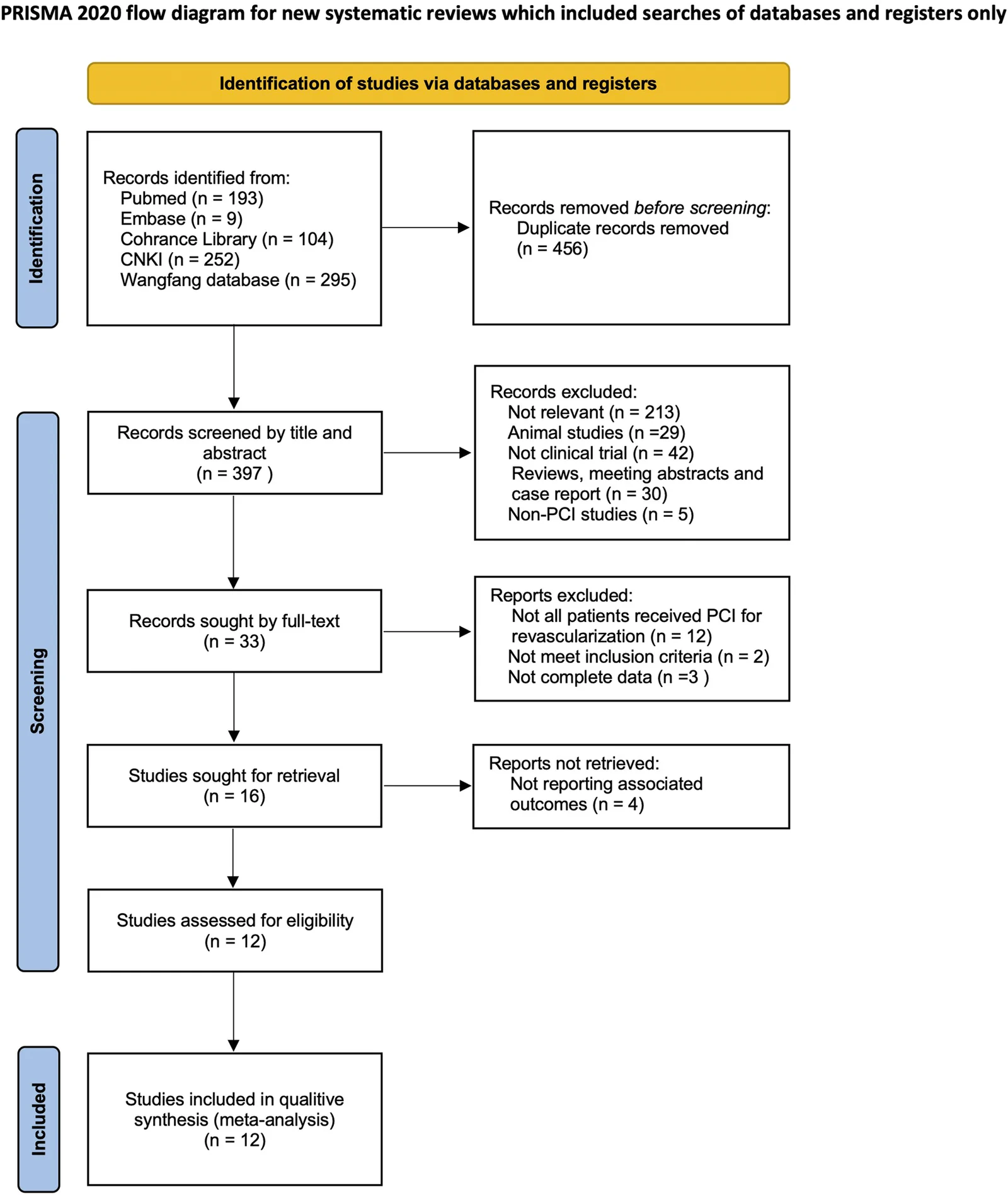
The PRISMA flow diagram of the literature search. PRISMA, Preferred Reporting Items for Systematic Reviews and Meta-Analyses; PCI, percutaneous coronary intervention; CNKI, China National Knowledge Infrastructure.

According to our search strategy, 1,153 records were identified. As shown in the PRISMA flow diagram (Figure 1), after removal of duplicates and screening of titles/abstracts and full texts, 12 studies involving 7,928 patients with CHD undergoing PCI that compared indobufen-with aspirin-based antiplatelet therapy were included in the final meta-analysis (Wu et al., 2023; Dai C. et al., 2024; Dai et al., 2025; Dai W. B. et al., 2024; Lin et al., 2020; Tu et al., 2021; Zhi, 2021; Yin et al., 2022; Feng et al., 2021; Zeng et al., 2020; Xiong and Zhang, 2020; Jiang et al., 2025; Wu et al., 2023). Among the 12 included studies, 9 were randomized controlled trials (RCTs) and 3 were retrospective observational studies. All patients with CHD included in the literature received dual antiplatelet therapy with indobufen or aspirin combined with P2Y_12_ receptor antagonists after PCI. Among them, 3,945 patients received indobufen antiplatelet treatment after PCI, while others only received aspirin antiplatelet treatment. Key study characteristics, including author, type of patients, participant characteristics (sex and age), interventions, and follow-up, are presented in Table 1. Furthermore, detailed baseline characteristics, study designs, endpoint definitions, and risk-of-bias ratings of the included studies are summarized in Supplementary Table S4. Notably, six studies enrolled only patients with ACS who underwent PCI and received dual antiplatelet therapy.

**TABLE 1:** Basic information of included studies.

Author	Type of patients	Gender (male/female)		Age		Intervening measures		Follow-up (months)
		T	C	T	C	T	C	
Lin F-2020 (Lin et al., 2020)	SAP	20/17	22/15	71.56 ± 2.19	71.66 ± 2.45	Clopidogrel 75 mg qd + Indobufen 100 mg bid	Clopidogrel 75 mg qd + Aspirin 100 mg qd	12
Zeng FF-2020 (Zeng et al., 2020)	CHD	17/13	16/14	73.30 ± 9.34	71.60 ± 8.96	Ticagrelor 90 mg bid + Indobufen 100 mg bid	Ticagrelor 90 mg bid + Aspirin 100 mg qd	6
Xiong LM-2020 (Xiong and Zhang, 2020)	AMI	70/36	66/40	53.02 ± 3.06	52.17 ± 3.27	Ticagrelor 90 mg bid + Indobufen 400 mg bid	Ticagrelor 90 mg bid + Aspirin 100 mg qd	3
Zhi WM-2021 (Zhi, 2021)	CHD	26/17	25/18	58.56 ± 6.04	58.63 ± 6.08	Clopidogrel 75 mg qd + Indobufen 100 mg bid	Clopidogrel 75 mg qd + Aspirin 100 mg qd	12
Yin XJ-2021 (Dai et al., 2025)	AMI	17/13	14/16	61.80 ± 5.10	62.20 ± 4.90	Clopidogrel 75 mg qd + Indobufen 100 mg bid	Clopidogrel 75 mg qd + Aspirin 100 mg qd	3
Feng YM-2021 (Feng et al., 2021)	AMI	49/27	51/28	56.97 ± 2.26	57.42 ± 2.29	Ticagrelor 90 mg bid + Indobufen 100 mg bid	Ticagrelor 90 mg bid + Aspirin 100 mg qd	1
Tu ZT-2021 (Tu et al., 2021)	AMI	30/23	31/22	65.30 ± 9.80	64.80 ± 10.20	Ticagrelor 90 mg bid + Indobufen 100 mg bid	Ticagrelor 90 mg bid + Aspirin 100 mg qd	6
Wu HY-2022 (Wu et al., 2023)	cTnI/T (−)CHD	1,521/737	1,447/846	61.00 ± 8.30	61.20 ± 8.40	Clopidogrel 75 mg qd + Indobufen 100 mg bid	Clopidogrel 75 mg qd + Aspirin 100 mg qd	12
Dai CF-2024 (Dai C. et al., 2024)	CHD	478/184	469/193	66.00 ± 9.40	66.00 ± 10.50	P2Y_12_ receptor antagonist + Indobufen 100 mg bid	P2Y_12_ receptor antagonist + Aspirin 100 mg qd	12
Dai WB-2024 (Dai W. B. et al., 2024)	AMI	141/83	150/74	72.00 ± 11.00	72.00 ± 10.00	Clopidogrel 75 mg qd + Indobufen 100 mg bid	Clopidogrel 75 mg qd + Aspirin 100 mg qd	12
Jiang ZH-2025 (Jiang et al., 2025)	CHD	63/57	68/52	68.50 ± 12.20	69.70 ± 10.20	Clopidogrel 75 mg qd + Indobufen 100 mg bid	Clopidogrel 75 mg qd + Aspirin 100 mg qd	12
Dai WB-2025 (Dai et al., 2025)	ACS	150/156	149/157	75.70 ± 7.05	75.80 ± 6.54	Clopidogrel 75 mg qd + Indobufen 100 mg bid	Clopidogrel 75 mg qd + Aspirin 100 mg qd	12

T, indobufen group; C, aspirin group; CHD, coronary heart disease; SAP, stable angina pectoris; AMI, acute myocardial infarction; ACS, acute coronary syndrome; cTnI/T (−), cardiac troponin I/T negative; qd, once daily; bid, twice daily.

### 3.2 Primary outcomes

#### 3.2.1 Incidence of MACE

**FIGURE 2:**
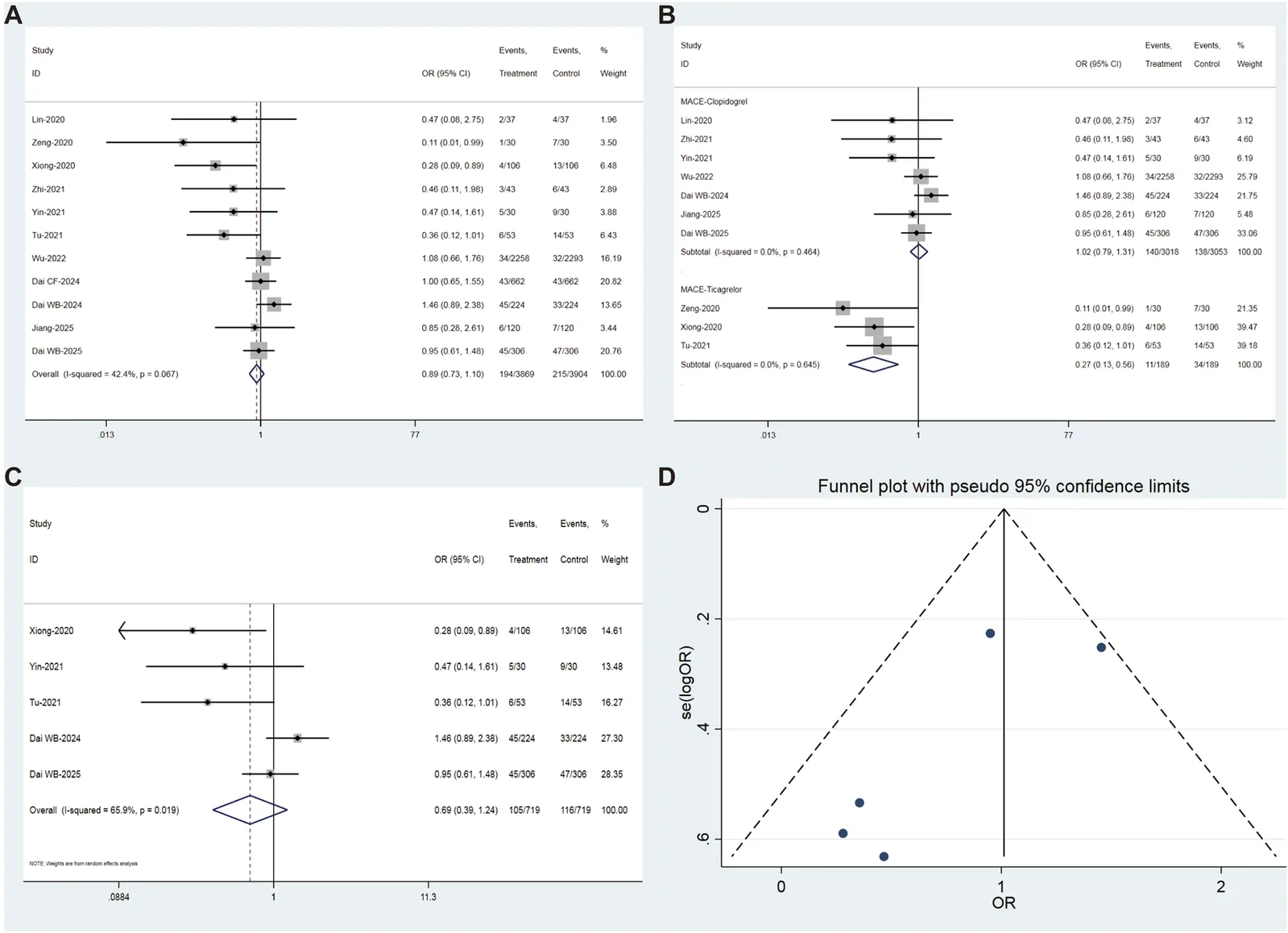
Meta-analysis of major adverse cardiac events (MACE) incidence comparing indobufen-versus aspirin-based dual antiplatelet therapy (DAPT). **(A)** Total population analysis using a fixed-effects model; **(B)** Subgroup analysis by concomitant P2Y_12_ inhibitor therapy using a fixed-effects model; **(C)** Risk of MACE in the acute coronary syndrome (ACS) subgroup using a random-effects model; **(D)** Funnel plot for the assessment of publication bias regarding the primary outcome. MACE, major adverse cardiac events; OR, indicates odds ratio; DAPT, dual antiplatelet therapy; ACS, acute coronary syndrome; CI, confidence interval.

Totally, 11 studies including 7,773 patients reported the incidence of MACE during follow-up. Relatively small heterogeneity was found (*I* ^2^ = 42.4%) and fixed effect model was used. As shown in Figure 2A, there was no significant difference in the incidence of MACE after PCI between the indobufen and aspirin groups (OR = 0.89, 95% CI 0.73–1.10, *P* = 0.284). However, a subgroup analysis based on study design revealed that in the RCT subgroup, indobufen was associated with a statistically significant reduction in MACE (OR = 0.65, 95% CI 0.46–0.90, *P* < 0.05, I^2^ = 35.4%), whereas no significant difference was observed in the non-RCT subgroup (OR = 1.09, 95% CI 0.84–1.42, P = 0.503, I^2^ = 0%) (Supplementary Figure S2A). We further performed subgroup analyses by P2Y_12_ inhibitor type. Compared with aspirin-based DAPT, the indobufen-based DAPT showed no significant difference in MACE when combined with clopidogrel (OR = 1.02, 95% CI 0.79–1.31, *P* = 0.873, *I* ^2^ = 0%). When combined with ticagrelor, the indobufen-based DAPT was associated with a lower risk of MACE during follow-up after PC (Figure 2B). In the ACS subgroup, indobufen was not associated with an increased risk of MACE after PCI compared with aspirin, with moderate heterogeneity (OR = 0.69, 95% CI 0.39–1.24, *P = 0.218*, *I* ^2^ = 65.9%) (Figure 2C). A random-effects model was employed due to the sources of heterogeneity. Furthermore, in the subgroup analysis based on follow-up duration, indobufen significantly reduced the risk of MACE in the 6 months follow up group (OR = 0.31, 95% CI 0.17–0.58, *P* < 0.001, I^2^ = 0%) whereas no statistically significant difference was observed in the 1-year follow-up subgroup (OR = 1.04, 95% CI 0.84–1.30, *P* = 0.714, I^2^ = 0%) (Supplementary Figure S3A). To assess potential publication bias, a funnel plot was generated (Figure 2D).

#### 3.2.2 Incidence of bleeding events

**FIGURE 3:**
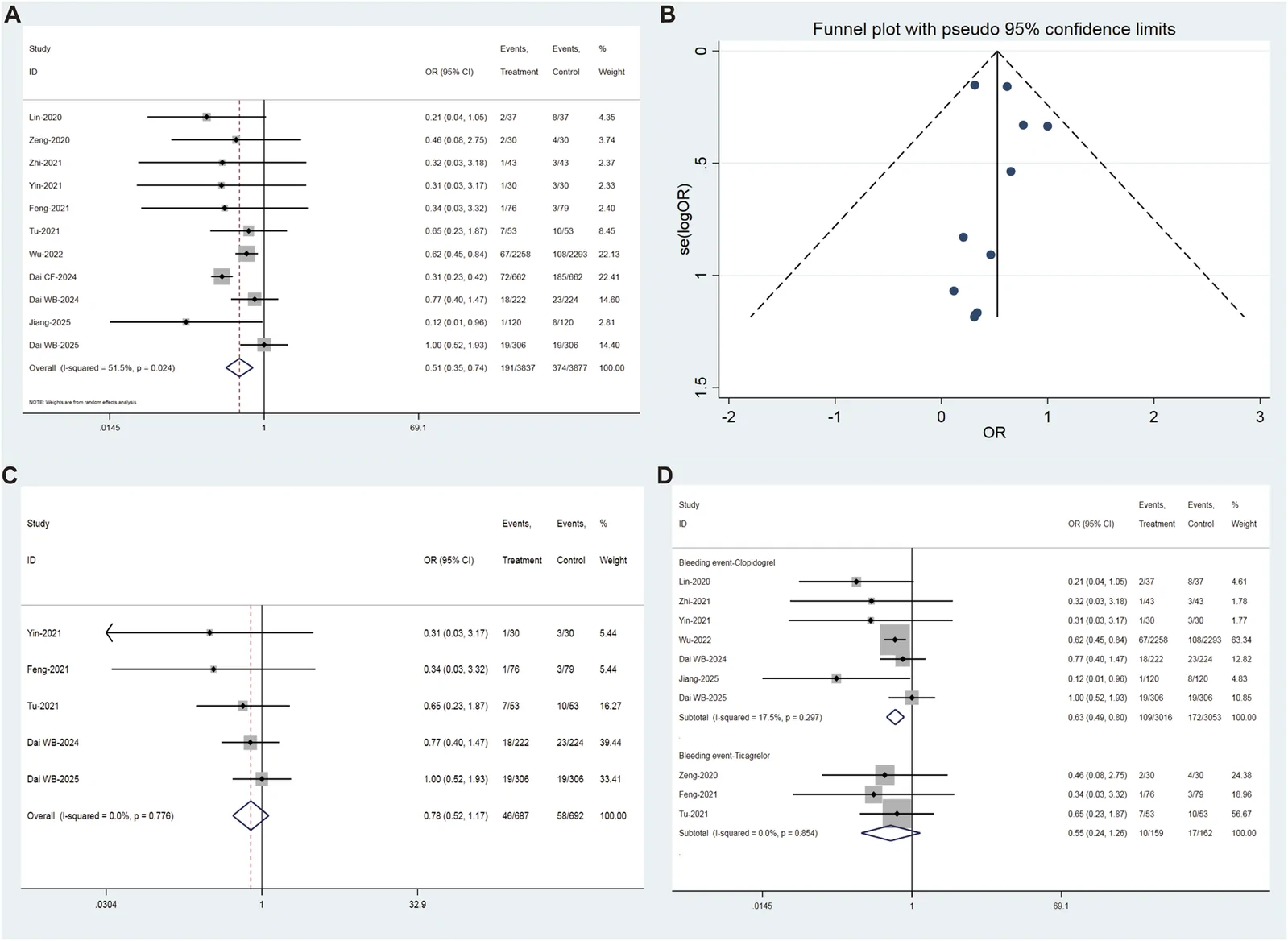
Meta-analysis of bleeding events comparing indobufen-versus aspirin-based dual antiplatelet therapy (DAPT). **(A)** Total population analysis of bleeding risk using a random-effects model; **(B)** Funnel plot for the assessment of publication bias regarding bleeding risk in the total population; **(C)** Risk of bleeding events in the acute coronary syndrome (ACS) subgroup using a fixed-effects model; **(D)** Subgroup analysis by concomitant P2Y_12_ inhibitor therapy using a fixed-effects model; DAPT, dual antiplatelet therapy; ACS, acute coronary syndrome; OR indicates odds ratio; CI, confidence interval.

Bleeding events were evaluated in 11 studies (including 7,716 patients). Our meta-analysis showed that, compared with aspirin, indobufen significantly reduced the risk of bleeding events in patients undergoing PCI, with moderate heterogeneity (OR = 0.51, 95% CI 0.35–0.74, *P* < 0.001, *I* ^2^ = 51.4%) (Figure 3A). Funnel plots are shown in Figure 3B to assess potential publication bias. Subgroup analysis by study design showed that indobufen significantly reduced bleeding in both the RCT subgroup (OR = 0.55, 95% CI 0.42–0.72, *P* < 0.001) and the non-RCT subgroup (OR = 0.42, 95% CI 0.33–0.54, *P* < 0.001). Notably, heterogeneity was completely eliminated in the RCT subgroup (I^2^ = 0%), whereas the non-RCT subgroup exhibited high heterogeneity (I^2^ = 85.4%) (Supplementary Figure S2E). In the ACS subgroup, indobufen was not associated with a statistically significant reduction in bleeding compared with aspirin (OR = 0.78, 95% CI 0.52–1.19, *P* = 0.229, *I* ^2^ = 0%) (Figure 3C). In the clopidogrel subgroup, indobufen-based DAPT significantly reduced bleeding compared with aspirin-based DAPT (OR = 0.63, 95% CI 0.49–0.80, *P* < 0.001, *I* ^2^ = 17.5%). By contrast, it did not lower the risk of bleeding during long-term treatment when used in combination with ticagrelor (OR = 0.55, 95% CI 0.24–1.26, *P* = 0.158, *I* ^2^ = 0%) (Figure 3D). Subgroup analysis by follow-up duration for bleeding events revealed no significant difference in the ≤6-month subgroup (OR = 0.51, 95% CI 0.23–1.12, P = 0.092, I^2^ = 0.0%), but a significant reduction in the 1-year subgroup (OR = 0.47, 95% CI 0.39–0.57, P < 0.001, I^2^ = 70.1%) (Supplementary Figure S3E).

### 3.3 Secondary outcomes

#### 3.3.1 Incidence of myocardial reinfarction, repeated revascularization and cardiovascular death

Furthermore, subgroup analyses based on study design (RCT vs. non-RCT) and follow-up duration demonstrated that the results for myocardial reinfarction, repeated revascularization, and cardiovascular death remained consistent across both subgroups, with no significant differences observed (all P > 0.05), which aligned strictly with the overall pooled analyses (Supplementary Figures S2B–D AND Supplementary Figures S3B–D).

**FIGURE 4:**
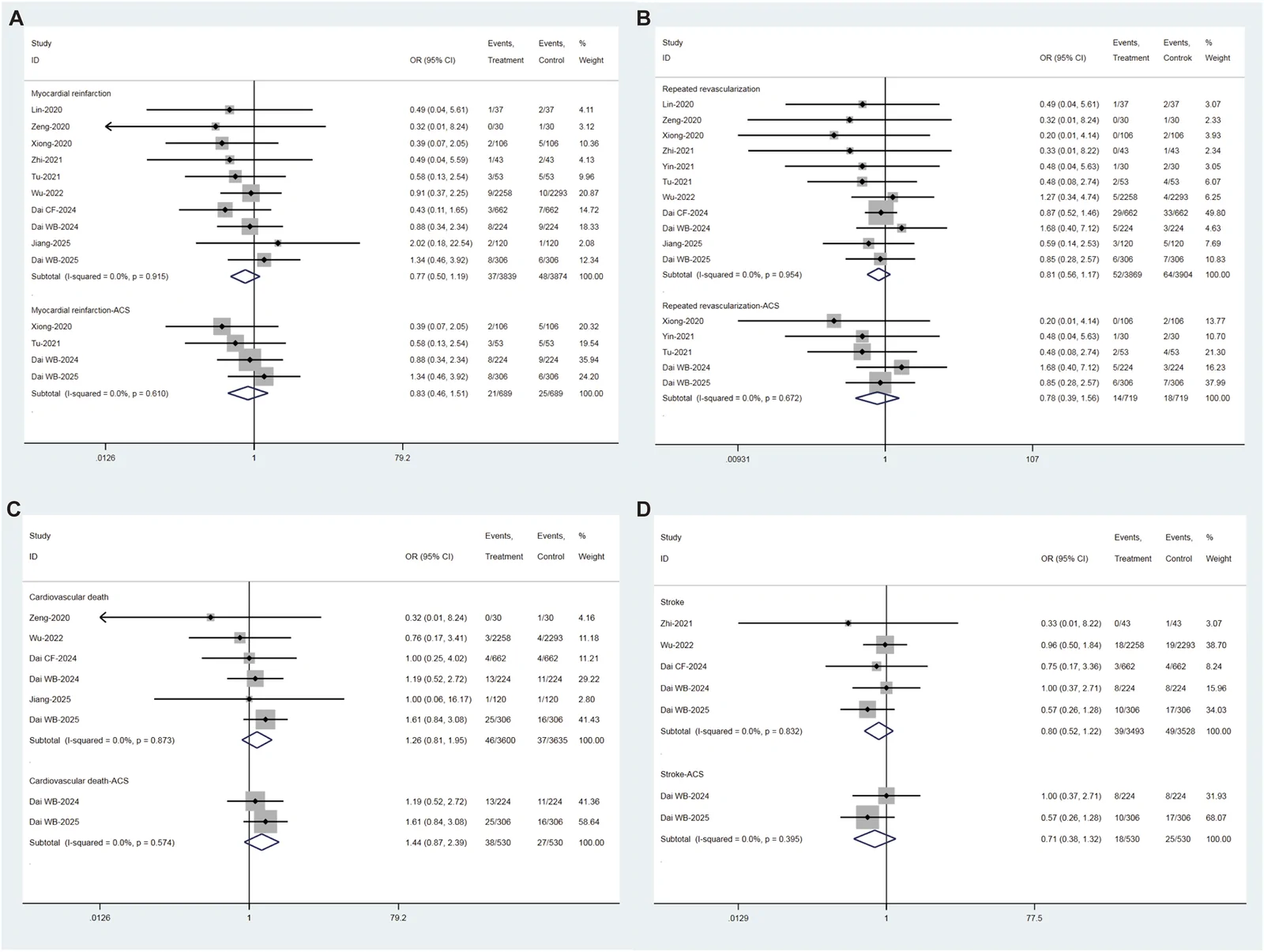
Meta-analysis of individual ischemic and cardiovascular events comparing indobufen-versus aspirin-based dual antiplatelet therapy (DAPT). **(A)** Risk of recurrent myocardial infarction in the total population and acute coronary syndrome (ACS) subgroup using a fixed-effects model; **(B)** Risk of repeat revascularization in the total population and ACS subgroup using a fixed-effects model; **(C)** Risk of cardiovascular death in the total population and ACS subgroup using a fixed-effects model; **(D)** Risk of stroke in the total population and ACS subgroup using a fixed-effects model. ACS indicates acute coronary syndrome; DAPT, dual antiplatelet therapy; ACS, acute coronary syndrome; OR, odds ratio; CI, confidence interval.

No significant heterogeneity was observed in the incidence of myocardial reinfarction, repeated revascularization and cardiovascular death (*I* ^*2*^ = 0%), whether in the population with CHD or the ACS subgroup, so a fixed effect model was used for meta-analysis. There were a total of 10 studies, including 7,713 patients, reporting the incidence of myocardial reinfarction, of which 4 studies focused on the ACS population. Compared with aspirin, indobufen was not associated with an increased risk of recurrent myocardial infarction after PCI in both the overall CHD population and the ACS subgroup (CHD: OR = 0.77, 95% CI 0.50–1.19, *P* = 0.237 and ACS: OR = 0.83, 95% CI 0.46–1.51, *P* = 0.548, Figure 4A).

11 studies with 7,773 patients explored the risk of repeated revascularization after PCI, including 5 studies on the ACS population. Similarly, compared with aspirin, indobufen was not associated with an increased risk of repeat revascularization after PCI in either the overall CHD population or the ACS subgroup (CHD: OR = 0.81, 95% CI 0.56–1.17, *P* = 0.266 and ACS: OR = 0.78, 95% CI 0.39–1.56, *P* = 0.481, Figure 4B).

6 studies involving 7,235 patients explored the incidence of cardiovascular death, including 2 studies on the ACS population. Compared to aspirin, indobufen does not increase the incidence of cardiovascular death during follow-up after PCI in both the overall CHD population and the ACS subgroup (CHD: OR = 1.26, 95% CI 0.81–1.95, *P* = 0.312 and ACS: OR = 1.44, 95% CI 0.87–2.39, *P* = 0.161, Figure 4C).

#### 3.3.2 Incidence of stroke

Stroke outcomes were reported in five studies involving 7,021 patients, including two studies in the ACS population. Compared with aspirin, indobufen was not associated with an increased risk of stroke after PCI in either the overall CHD population or the ACS subgroup, with no heterogeneity observed (CHD: OR = 0.80, 95% CI 0.52–1.22, *P =* 0.298*, I* ^*2*^ = 0% and ACS: OR = 0.71, 95% CI 0.38–1.32, *P =* 0.278*, I* ^*2*^ = 0%, Figure 4D).

#### 3.3.3 Incidence of gastrointestinal symptom

**FIGURE 5:**
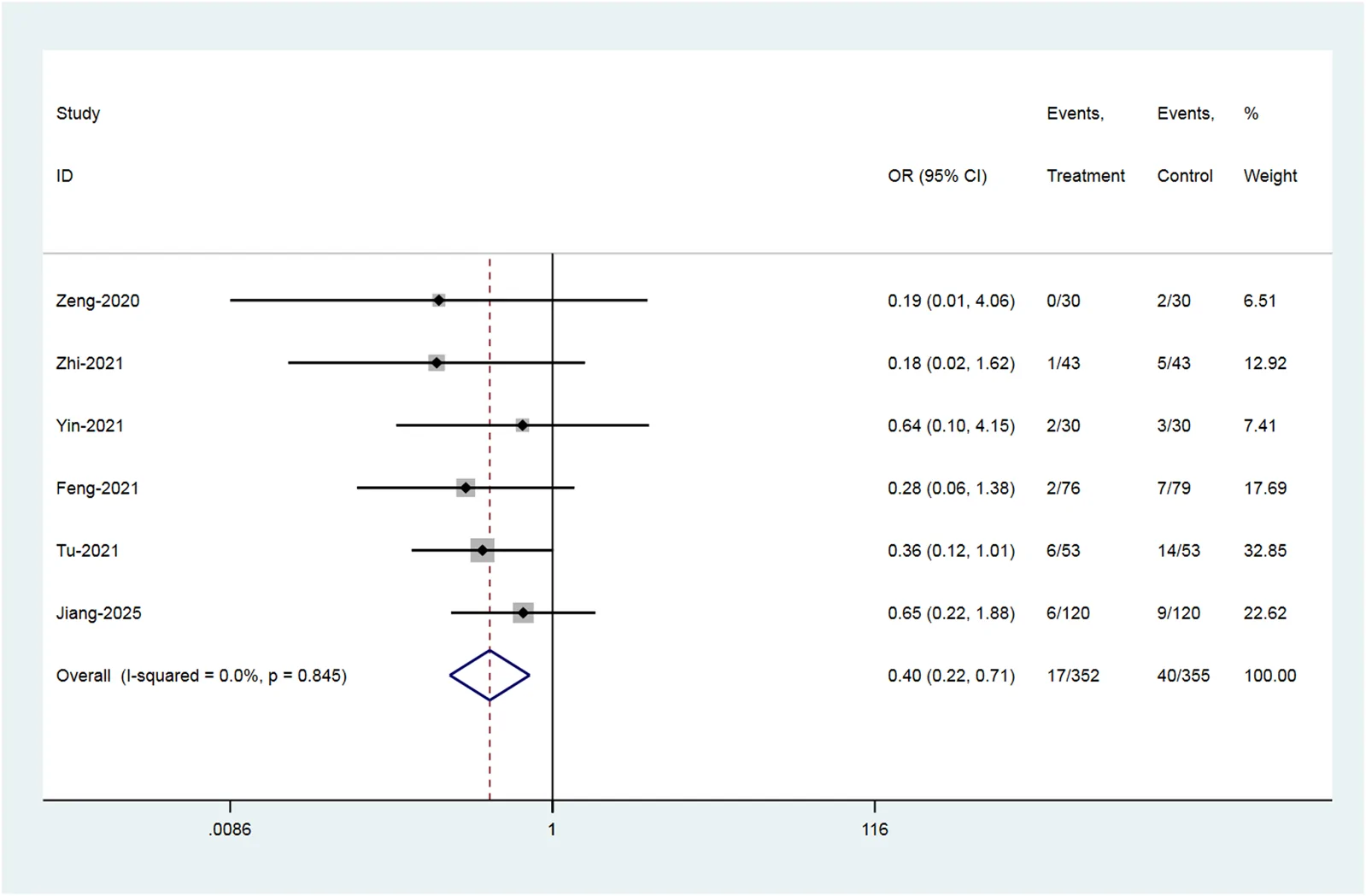
Meta-analysis of gastrointestinal symptoms comparing indobufen-versus aspirin-based dual antiplatelet therapy (DAPT) using a fixed-effects model. DAPT, dual antiplatelet therapy; OR indicates odds ratio; CI, confidence interval.

Gastrointestinal symptoms were evaluated in 6 studies (including 707 patients). The use of indobufen during DAPT after PCI was associated with a lower rate of gastrointestinal symptoms compared with aspirin (OR = 0.40, 95% CI 0.22–0.71, *P* < 0.01, *I* ^2^ = 0%) (Figure 5). However, specific data on gastrointestinal symptoms for the ACS subgroup were unavailable.

## 4 Discussion

This meta-analysis suggests that, in patients receiving DAPT after PCI, replacing aspirin with indobufen provides comparable protection against MACE without increasing recurrent MI, repeat revascularization, or cardiac death, while reducing bleeding and gastrointestinal adverse events. However, within the ACS subgroup, there were no significant differences between indobufen and aspirin regarding either ischemic outcomes or bleeding events, Notably, the pooled MACE analysis for this subset exhibited moderate heterogeneity (I^2^ = 65.9%), which likely stems from inherent clinical variations across the five included trials, such as differences in baseline patient acuity and follow-up durations. Furthermore, specific data on gastrointestinal symptoms were unavailable.

In patients with ACS and/or undergoing PCI, DAPT with aspirin and a P2Y_12_ receptor antagonist is the cornerstone of treatment. DAPT was required in patients undergoing PCI with drug-eluting stents (DES) or bare-metal stents (BMS) for it could evidently reduce the risk of cardiovascular death, spontaneous MI, stroke and MACE (Steinhubl et al., 2002). However, aspirin intolerance may compromise adherence to DAPT and lead to premature discontinuation, thereby increasing ischemic risk. In the SYMPHONY I and II trials, gastrointestinal discomfort was reported in 11.9% of participants (Newby et al., 2003). Furthermore, studies focusing on Chinese populations undergoing PCI have indicated that the incidence of aspirin intolerance exceeds 10% following the procedure (Dai et al., 2020). Aspirin intolerance following PCI is associated with reduced adherence to DAPT and may result in its premature discontinuation, consequently elevating the risk of ischemic events.

In clinical practice, the management of aspirin intolerance after PCI typically involves one of the following four strategies, each supported by varying levels of evidence and guideline recommendations.

First, proton pump inhibitors (PPIs) can be used concomitantly with aspirin. This approach is supported by ESC guidelines to reduce bleeding complications in high-risk patients (Byrne et al., 2023). However, long-term use of PPIs may carry potential adverse effects (Zheng et al., 2023; Meijvis et al., 2011; Cheung et al., 2018). Second, aspirin desensitization can be performed to induce temporary tolerance under controlled settings (Woessner, 2015). This approach involves the gradual administration of increasing doses of aspirin, usually starting at very low doses and escalating until a full therapeutic dose is tolerated. This strategy is particularly relevant in patients with a history of hypersensitivity reaction and is consistent with recommendations from allergy and immunology societies. However, this approach takes a relatively long time, it is not feasible for most patients with ACS, as they necessitate prompt aspirin therapy. Third, under the premise of fully assessing the risk of bleeding and ischemia, P2Y_12_ inhibitor monotherapy may be considered after PCI, particularly in patients who cannot tolerate DAPT or have a very high risk of bleeding, as endorsed by recent ESC guidelines (Byrne et al., 2023). At last, replacing aspirin with another antiplatelet drug is a common approach, especially in cases of persistent intolerance or desensitization failure. Cilostazol is a phosphodiesterase III inhibitor that inhibits platelet aggregation by increasing the concentration of cAMP within platelets. There is strong evidence of its use in east Asia (especially Japan and South Korea), but it is less mentioned in European and American guidelines (Nakamura et al., 2020).

Indobufen, a reversible COX inhibitor, is another choice for aspirin intolerence. Recent studies have reported that the antiplatelet effect of 100 mg twice daily dose of indobufen is bioequivalence with 100 mg once daily dose of aspirin (Shi et al., 2022). For patients with ischemic diseases requiring antiplatelet therapy, does indobufen provide equivalent antiplatelet value compared to aspirin? In patients with acute moderate-to-severe ischaemic stroke, indobufen was associated with a higher risk of recurrent stroke at 90 days compared to aspirin in those population (Pan et al., 2023), indicating bioequivalence or similar pharmacodynamic effects does not necessarily translate to equivalent clinical efficacy. However, evidence from the OPTION trial supports the use of indobufen, which has demonstrated non-inferiority to aspirin in reducing ischemic events with a lower bleeding risk in troponin-negative CHD patients after PCI (Wu et al., 2023).

Previous studies have shown that indobufen inhibits platelet aggregation through multiple mechanisms (Li et al., 2021). Similar to aspirin, indobufen could inhibit the synthesis of TXA2 and aggregation of platelets through inhibiting COX-1. In addition, it inhibits thrombus formation by preventing platelet aggregation through the arachidonic acid and adenosine diphosphate pathways (Wiseman et al., 1992). And the antiplatelet efficacy could be monitored and recorded as platelet aggregation rate (PAR) which had been deemed as an indicator for ischemic events in patients with ACS and with stable CHD (Breet et al., 2010). Recent study found that in comparison with aspirin, indobufen could significantly decrease PAR (Shi et al., 2022). It can be easily explained by the reversible inhibition of COX-1 without affecting prostacyclin production for indobufen. The effect was robust but transient.

Previous studies indicate that indobufen and standard dose aspirin have basically the same effect whether in biochemical, functional, and clinical effects by inhibiting COX-1 when working alone. There is no consensus on the effectiveness of indobufen in DAPT. In an experimental study, Li and his colleagues found that indobufen plus clopidogrel worked better on ADP induced platelet aggregation than aspirin plus clopidogrel (Li et al., 2021), while Barillà and his colleagues pointed that clopidogrel 75 mg daily plus indobufen 100 mg twice a day did not improve the inhibition rate of ADP-induced platelet aggregation compared with using clopidogrel 75 mg daily alone (Barilla et al., 2013). Ours indicated that replacing aspirin with indobufen in DAPT, which is used jointly with P2Y_12_, can still effectively reduce the incidence of MACE after PCI. The ASPIRATION registry study was the first real-world investigation to include the entire CHD population and to evaluate the efficacy and safety of a DAPT regimen containing indobufen, which aligns with our research findings (Dai C. et al., 2024).

Clopidogrel and ticagrelor are standard P2Y_12_ inhibitors in the currently recommended DAPT regimen. According to previous studies, different type of P2Y_12_ act totally different. In the PLATO trial, ticagrelor can lower the rate of MACE in ACS patients when compared to clopidogrel, the lower rate was 16% (Cannon et al., 2010). To minimize the heterogeneity related to different types of P2Y_12_ inhibitors, we conducted a subgroup analysis based on the type of P2Y_12_ inhibitor combined with indobufen after PCI, which demonstrated comparable efficacy on reducing the risk of MACE between indobufen and aspirin in the clopidogrel subgroup. Surprisingly, among patients receiving ticagrelor, indobufen suggested a potential benefit in reducing the risk of MACE after PCI versus aspirin.

Bleeding represents a common complication associated with DAPT according to some trials (Natsuaki et al., 2019). Even in combination with PPIs, bleeding events may still occur, potentially leading to treatment discontinuation and subsequent adverse cardiovascular outcomes. Indobufen exhibits several advantageous pharmacological properties, such as a rapid onset of action, reversible inhibition of platelet function, and a short metabolic half-life after drug withdrawal. Findings from prior research demonstrate that the risk of bleeding and gastrointestinal reactions is lower with indobufen compared to other antiplatelet agents (Shi et al., 2022). Although current ESC and AHA/ACC guidelines on CHD have not yet included indobufen as a recommended antiplatelet agent in post-PCI DAPT regimens, findings from current study reaffirmed that, compared with aspirin, indobufen significantly reduces the incidence of bleeding events and gastrointestinal adverse reactions, which is consistent with previous studies and highlighting its favorable safety profile. Given that the *I* ^*2*^ test value for risk of bleeding events (51.4%) is very close to 50% threshold and included studies were mostly symmetrically distributed in the funnel plot, the observed heterogeneity is considered acceptable. Our subgroup analysis stratified by study design (RCT vs. non-RCT) could identified the heterogeneity, with the I^2^ dropping to 0% within the RCT subgroup. This indicates that the observed heterogeneity was primarily driven by the methodological differences of non-randomized observational studies, which inherently carry a higher risk of selection and confounding biases compared to randomized trials. By contrast, management guidelines for non ST segment elevation myocardial infarction and chronic coronary syndrome in China offer clear recommendations for the use of indobufen. This endorsement is based primarily on its association with a lower risk of bleeding and fewer gastrointestinal side effects, particularly among patients with aspirin intolerance or elevated bleeding risk. It is worth noting that in the ACS subgroup analysis, the reduction in bleeding events did not reach statistical significance which might due to limited statistical power and the use of intensive concurrent antithrombotic therapies in acute settings. These findings may be linked to the acute stress state associated with ACS. Under such physiological stress, patients are more susceptible to stress-related gastric mucosal injury, which may subsequently predispose them to gastrointestinal bleeding (Young et al., 2024; Huang et al., 2013). Consequently, the intrinsic safety advantage of indobufen in reducing bleeding might be attenuated or masked by these overwhelming acute stress factors.

Our study also has some limitations. First, the included articles are mostly carried out in China. Although our comprehensive literature search was not restricted by region, this geographic concentration inevitably limits the generalizability of our results to other populations, as underlying genetic backgrounds and regional clinical practices may differ. Therefore, future large-scale, multi-national trials are necessary to validate these findings globally. Secondly, the follow-up duration across the studies included was unequal, and as the longest period was limited to 1 year. So, it was not possible to ascertain the long-term safety and efficacy profile of indobufen in CHD patients receiving DAPT after PCI. Third, due to the inclusion of retrospective studies in our meta-analysis which would results in inherent limitations such as selection bias, information bias, and confounding bias, which may reduce the reliability of the findings. Fourth, due to the lack of subgroup reporting in the original trials, we were unable to assess the incidence of gastrointestinal adverse events specifically within the ACS population.

In summary, this meta-analysis showed that, compared with aspirin, indobufen combined with a P2Y_12_ receptor antagonist was associated with a similar risk of MACE after PCI while also associated with a lower risk of bleeding and gastrointestinal discomfort in the overall CHD population. However, the current evidence is limited by the small number of studies, variable methodological quality, and heterogeneity; therefore, well-designed, adequately powered prospective RCTs are needed to confirm these findings.

## Data Availability

The original contributions presented in the study are included in the article/Supplementary Material, further inquiries can be directed to the corresponding author.

## Supplementary material

SUPPLEMENTARY FIGURE S1 Risk of bias assessment of the included studies using the Cochrane Risk of Bias 2 (RoB 2) tool. **(A)** Risk of bias graph: review authors’ judgements about each risk of bias domain presented as percentages across all included studies. **(B)** Risk of bias summary: review authors’ judgements about each risk of bias domain for each individual study. Green circles with a plus sign (+) indicate low risk of bias; yellow circles with a minus sign (−) indicate some concerns. Abbreviations: D1, bias arising from the randomization process; D2, bias due to deviations from intended interventions; D3, bias due to missing outcome data; D4, bias due to outcome measurement; D5, bias due to selection of the reported result.

SUPPLEMENTARY FIGURE S2 Subgroup analyses of primary and secondary outcomes based on study design (RCT vs. non-RCT). Forest plots comparing indobufen-versus aspirin-based dual antiplatelet therapy (DAPT) stratified by randomized controlled trials (RCTs) and non-RCTs for: **(A)** Major adverse cardiac events (MACE); **(B)** Myocardial reinfarction; **(C)** Repeated revascularization; **(D)** Cardiovascular death and **(E)** Bleeding events. RCT, randomized controlled trial; OR, odds ratio; CI, confidence interval; MACE, major adverse cardiac events.

SUPPLEMENTARY FIGURE S3 Subgroup analyses of follow-up duration (≤6 months vs. 1 year). Forest plots comparing indobufen-versus aspirin-based dual antiplatelet therapy (DAPT) stratified by randomized controlled trials (RCTs) and non-RCTs for: **(A)** Major adverse cardiac events (MACE); **(B)** Myocardial reinfarction; **(C)** Repeated revascularization; **(D)** Cardiovascular death and **(E)** Bleeding events. OR, odds ratio; CI, confidence interval; MACE, major adverse cardiac events.
