# Correction: Indobufen-based dual antiplatelet therapy in patients undergoing PCI: a systematic review and meta-analysis

**DOI:** 10.3389/fphar.2026.1966715

**Published:** 2026-09-21

**Authors:** Yiheng Liu, Bin Liu, Weiran Dai

**Affiliations:** 1 Department of Cardiology, The Second Affiliated Hospital of Chongqing Medical University, Chongqing, China; 2 Department of Cardiology, West China Hospital, Sichuan University, Chengdu, China

**Keywords:** aspirin, cardiovascular outcomes, dual antiplatelet therapy, indobufen, major adverse cardiac events, meta-analysis

Authors Yiheng Liu and Bin Liu were erroneously omitted as equal contributing authors.

The original version of this article has been updated.

