# Supplementary material for: Indobufen-based dual antiplatelet therapy in patients undergoing PCI: a systematic review and meta-analysis
Source: Front Pharmacol. 2026 Jul 24;17:1821883. doi: 10.3389/fphar.2026.1821883 (PMC13447155; doi:10.3389/fphar.2026.1821883)
Supplement: Supplementary file 1 [file Table1.docx]

**Table S1. Detailed search strategy used in the systematic review and meta-analysis**

| Concept | Search terms |
| --- | --- |
| Intervention: Indobufen | “indobufen” OR “Ibustrin” |
| Population: PCI/CAD | “percutaneous coronary intervention” OR “PCI” OR “coronary heart disease” OR “CHD” OR “myocardial infarction” OR “MI” OR “acute myocardial infarction” OR “AMI” OR “acute coronary syndrome” OR “ACS” |
| Final Boolean strategy | (“indobufen” OR “Ibustrin”) AND (“percutaneous coronary intervention” OR “PCI” OR “coronary heart disease” OR “CHD” OR “myocardial infarction” OR “MI” OR “acute myocardial infarction” OR “AMI” OR “acute coronary syndrome” OR “ACS”) |

**Abbreviations**: PCI=percutaneous coronary intervention; CAD=coronary artery disease; CHD=coronary heart disease; MI=myocardial infarction; AMI=acute myocardial infarction; ACS=acute coronary syndrome;

**Table S2. Methodological quality of included retrospective studies based on the Newcastle-Ottawa Scale (NOS)**

| Study (Year) | Selection | Comparability | Outcome | Total Score | Quality |
| --- | --- | --- | --- | --- | --- |
| Dai CF-2024 | ★★★★ | ★★ | ★★ | 8 | High |
| Dai WB-2025 | ★★★★ | ★★ | ★★★ | 9 | High |
| Dai WB-2024 | ★★★★ | ★★ | ★ | 7 | High |

**Table S3. The specific definitions of Endpoint Definitions (MACE, bleeding, and gastrointestinal symptoms) for each included study.**

| Study | MACE Detailed Definition | Bleeding Detailed Definition | GI Symptoms Detailed Definition |
| --- | --- | --- | --- |
| Lin F-2020 | CV death, MI, TVR | major and minor bleeding | gastrointestinal bleeding/melena |
| Zeng FF-2020 | Angina, MI, cardiac death, stroke, stent thrombosis | Gastrointestinal and intracranial bleeding | Gastrointestinal bleeding (reported under bleeding) |
| Xiong LM-2020 | MI, arrhythmia, TVR, angina | Not reported | Not reported |
| Zhi WM-2021 | CV death, MI, stroke, TVR | Gums, subcutaneous, hematuria, and GI bleeding | Nausea, vomiting, abdominal pain, diarrhea |
| Yin XJ-2021 | Angina, ECG changes, acute CV events, stent thrombosis | Fecal occult blood (reported under gastrointestinal risk) | Abdominal pain, fecal occult blood |
| Feng YM-2021 | Not reported | Gum bleeding, melena (reported under adverse reactions) | Nausea, vomiting (reported under adverse reactions) |
| Tu ZT-2021 | Cardiac death, recurrent MI, TVR, malignant arrhythmia | Severe, minor, and mild bleeding (PLATO[13]) | Gastrointestinal reactions |
| Wu HY-2022 | CV death, nonfatal MI, ischemic stroke, definite/probable ST | BARC [14] type 2, 3, or 5 bleeding | Gastrointestinal bleeding (reported under BARC bleeding) |
| Dai CF-2024 | CV death, nonfatal MI, repeated revascularization, definite/probable ST, nonfatal ischemic stroke (referred to as MACCE) | Bleeding events according to BARC criteria type 2, 3, or 5 | Gastrointestinal bleeding (reported under BARC bleeding) |
| Dai WB-2024 | Cardiac death, recurrence of MI, definite/probable ST, TLR, and ischemic stroke (reported as parts of NACE) | Bleeding events according to BARC criteria type 2, 3, or 5 | Gastrointestinal bleeding (reported under BARC bleeding) |
| Jiang ZH-2025 | Target vessel MI, cardiac death, and TLR | Bleeding according to BARC criteria | Gastrointestinal bleeding |
| Dai WB-2025 | All-cause death, stroke, MI, and TLR (reported as parts of NACE) | Bleeding events according to BARC criteria type 2, 3, or 5 | Gastrointestinal reactions/events (reported as safety outcome) |

**Abbreviations**: BARC=Bleeding Academic Research Consortium; CV=cardiovascular; ECG=electrocardiogram; GI=gastrointestinal; MACCE=major adverse cardiac and cerebrovascular events; MACE=major adverse cardiovascular events; MI=myocardial infarction; NACE=net adverse clinical events; PLATO=Platelet Inhibition and Patient Outcomes; ST=stent thrombosis; TLR=target lesion revascularization; TVR=target vessel revascularization.

**Table S4. Detailed baseline characteristics and methodology of the included studies.**

| Study | Design | Country | Inclusion Criteria | PCI Context | ACS Status | Antiplatelet Regimens | Indobufen Dose | Follow-up Duration |
| --- | --- | --- | --- | --- | --- | --- | --- | --- |
| Lin F-2020 | RCT | China | SAP, post-PCI | PCI | Non-ACS (SAP) | T: Clopidogrel + Indobufen  C: Clopidogrel + Aspirin | 200 mg/d (or 100 mg bid) | 12 months |
| Zeng FF-2020 | Retrospective cohort | China | CHD, post-PCI | PCI | CHD | T: Ticagrelor + Indobufen  C: Ticagrelor + Aspirin | 200 mg/d (or 100 mg bid) | 6 months |
| Xiong LM-2020 | RCT | China | AMI, post-PCI | PCI | AMI | T: Ticagrelor + Indobufen  C: Ticagrelor + Aspirin | 0.4 g bid (800 mg/d) | 3 months |
| Zhi WM-2021 | RCT | China | CHD, post-PCI | PCI | CHD | T: Clopidogrel + Indobufen  C: Clopidogrel + Aspirin | 200 mg/d (100 mg bid) | 12 month |
| Yin XJ-2021 | RCT | China | AMI, post-PCI | PCI | AMI | T: Clopidogrel + Indobufen  C: Clopidogrel + Aspirin | 200 mg/d (100 mg bid) | 3 months |
| Feng YM-2021 | RCT | China | AMI, post-PCI | PCI | AMI | T: Ticagrelor + Indobufen  C: Ticagrelor + Aspirin | 100 mg bid | 1 month |
| Tu ZT-2021 | RCT | China | AMI, post-PCI | PCI | ACS (STEMI) | T: Ticagrelor + Indobufen  C: Ticagrelor + Aspirin | 100 mg bid | 6 months |
| Wu HY-2022 | RCT | China | cTnI/T（-）CHD, post PCI | PCI (DES) | CCS / stabilized ACS | T: Clopidogrel + Indobufen  C: Clopidogrel + Aspirin | 200 mg/d (100 mg bid) | 12 months |
| Dai CF-2024 | Retrospective cohort | China | CHD, post-PCI | PCI | CHD | T: P2Y12 inhibitor + Indobufen  C: P2Y12 inhibitor + Aspirin | 100 mg bid (200 mg/d) | 12 months |
| Dai WB-2024 | Retrospective cohort | China | AMI, post-PCI | PCI | ACS (STEMI and NSTEMI) | T: Clopidogrel + Indobufen  C: Clopidogrel + Aspirin | 100 mg bid (200 mg/d) | 12 months |
| Jiang ZH-2025 | RCT | China | CHD, post PCI | PCI (with DEB) | CHD | T: Clopidogrel + Indobufen C: Clopidogrel + Aspirin | 100 mg bid (200 mg/d) | 12 months |
| Dai WB-2025 | Retrospective cohort | China | ACS, post-PCI | PCI (DES or DEB) | ACS (UA, NSTEMI, STEMI) | T: Clopidogrel + Indobufen  C: Clopidogrel + Aspirin | 100 mg bid (200 mg/d) | 12 months |

**Abbreviations**:ACS=acute coronary syndrome; AMI=acute myocardial infarction; bid=twice daily; C=control; CAD=coronary artery disease; CCS=chronic coronary syndrome; CHD=coronary heart disease; DEB=drug-coated balloon; DES=drug-eluting stent; GI=gastrointestinal; NSTEMI=non-ST-segment elevation myocardial infarction; PCI=percutaneous coronary intervention; RCT=randomized controlled trial; SAP=stable angina pectoris; STEMI=ST-segment elevation myocardial infarction; T=treatment; UA=unstable angina.
